# Schisandrin B Alleviates Lipid Metabolism Disorders and Apoptosis of MAFLD via Modulation of PPARγ-PCK1 and Caspase-3 Signaling Pathways

**DOI:** 10.3390/ph18101441

**Published:** 2025-09-25

**Authors:** Meng Gao, Feilong Liu, Xiyuan Feng, Mengyang Wang, Zhihong Zhang, He Li, Chunmei Wang, Jinghui Sun

**Affiliations:** School of Pharmacy, Beihua University, Jilin 132013, China; s9912062636@163.com (M.G.); m18831442942@163.com (F.L.); 18004369287@163.com (X.F.); 15643584357@163.com (M.W.); zhzhang518@163.com (Z.Z.); yitonglh@126.com (H.L.); wangcm74@126.com (C.W.)

**Keywords:** Schisandrin B, peroxisome proliferator-activated receptor gamma, metabolic-associated fatty liver disease

## Abstract

**Objectives**: This study focuses on the regulatory mechanism of Schisandrin B (Sch B) on the lipid metabolism and apoptosis of AML-12 liver cells, with a particular emphasis on its potential therapeutic effect and mechanism of action in preventing and treating metabolic-associated fatty liver disease (MAFLD) by activating the PPARγ signaling pathway. **Methods**: An MAFLD cell model was established by inducing AML-12 cells with a mixture of oleic acid (OA) and palmitic acid (PA) (2:1). AML-12 cells were divided into a control group, a model group, and 20 μM and 40 μM Sch B groups. The cells were lysed and prepared into the cell suspension, then the cell suspension was centrifuged to obtain its supernatant, and the levels of total cholesterol (TC), triglycerides (TG), alanine aminotransferase (ALT), and aspartate aminotransferase (AST) in the supernatant were detected according to the instructions of the kits. Effects of Sch B on the pathological changes of AML-12 cells were observed by Oil Red O staining. The key targets were screened through network pharmacology, and relevant targets were verified through molecular docking simulation. The activity of PPARγ was detected using a dual luciferase reporter plasmid, and the level of cell apoptosis was detected using the Annexin V-FITC/PI double staining method. The Western blot method was used to analyze the expression of genes related to lipid metabolism and apoptosis pathways. **Results**: Sch B could regulate lipid metabolism disorders in OA+PA-induced MAFLD cell model. The activation of PPARγ-PCK1/Aspase is a key step in the action of Sch B, which can effectively block fatty acid synthesis, improve fatty acid oxidation, and reduce lipid droplet aggregation in liver cells, thereby alleviating lipid metabolism abnormalities in the MAFLD cell model and inhibiting cell apoptosis. **Conclusions**: This finding may lay an important theoretical foundation and open a new research direction for the deep development and application of *Schisandra chinensis*.

## 1. Introduction

Metabolic-associated fatty liver disease (MAFLD), formerly known as nonalcoholic fatty liver disease (NAFLD), is one of the most common chronic liver diseases in the world, and it has become the second major cause of end-stage liver disease in recent decades [1]. MAFLD is closely related to obesity, metabolic syndrome, type II diabetes, and so on [2]. MAFLD can not only lead to non-alcoholic fatty liver disease, cirrhosis, and even hepatocellular carcinoma, but also increase the mortality rate of liver-related diseases [3]. As of 2020, the global morbidity of MAFLD has reached 25% [4]. Due to the lack of approved specific drugs for treating MAFLD, dietary control and exercise are still the preferred treatment methods recommended by most scientific associations. However, these methods are not always sustainable and reliable.

Schisandrin B (Sch B) is an active lignan component in *Schisandra chinensis*, a plant in Magnoliaceae, and is widely used due to its prevention and treatment of liver diseases, and blood lipid-lowering and antioxidation effects [5,6,7]. Previous studies have confirmed that Sch B has a significant alleviating effect on MAFLD animal models, but its underlying mechanism is still unclear [8,9,10]. Peroxisome proliferator-activated receptor (PPAR), a member of the nuclear receptor family, plays a crucial role in maintaining metabolic homeostasis. Currently, there are three subtypes of PPAR, namely PPARα, PPARβ/δ, and PPARγ. PPAR can serve as a sensor for fatty acids, regulating various pathways involved in lipid and glucose metabolism, as well as energy metabolism [11,12]. Its role in liver injury has been repeatedly confirmed, and it is also an effective target for treating a variety of metabolic diseases, including type II diabetes and hyperlipidemia [13,14,15,16]. At present, it has been confirmed that Sch B has a definite liver protection and anti-inflammatory potential, and it can alleviate liver fibrosis induced by carbon tetrachloride through various regulatory mechanisms [17]. In addition, Sch B can effectively inhibit the inflammatory response of microglia induced by lipopolysaccharide (LPS) by activating the PPAR-γ signaling pathway [18]. However, there is still a lack of research on the specific role and molecular mechanism of Sch B in MAFLD, so this research gap provides us with new research directions to explore the potential value of Sch B in MAFLD treatment and explore its new regulatory targets.

Our team discovered through network pharmacology and molecular docking technology that the key target of *Schisandra chinensis* for treating MAFLD was PPARγ [19], which was verified in a high-fat diet-induced C57BL/6 mouse model. It was found that SchB could alleviate MAFLD in mice by regulating the PPARγ signaling pathway and gut microbiota [20]. On this basis, experiments around two innovative points were conducted in this study. On the one hand, AML-12 mouse liver cell lines were used for in vitro verification, which served as a key supplement to previous in vivo studies on a high-fat diet-induced mouse model to explore not only the protective and therapeutic effects of Sch B on the cells, but also the molecular mechanisms of action of Sch B at the cellular level. On the other hand, the focus of mechanism research could be further expanded from PPARγ to PCK1 and Caspase-3, and the core targets of Sch B therapy for MAFLD could be screened out through network pharmacology; then, molecular docking simulations, luciferase reporter plasmid experiments, and Western blot were used to verify whether Sch B would regulate lipid metabolism disorders through PPARγ, and simultaneously, flow cytometry was used to detect its effect on cell apoptosis, so as to clarify the multidimensional regulatory role of Sch B in the pathogenesis of MAFLD. This “in vitro–in vivo” progressive progress can not only provide a more comprehensive insight into the therapeutic potential of Sch B, but also a theoretical basis for further research, development, and application of Sch B.

## 2. Results

### 2.1. Effects of Different Induction Times on MAFLD Cell Model

**Figure 1:**
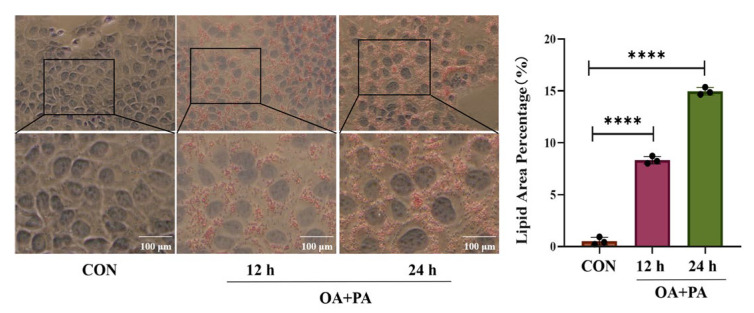
Effects of the induction at different times on MAFLD cell model (mean ± *S.E.*, *n* = 3). **** *p* < 0.0001 vs. MOD.

An MAFLD cell model was established by inducing AML-12 cells with oleic acid (OA) and palmitic acid (PA) at a ratio of 2:1 for 12 h and 24 h, respectively. As shown in Figure 1, compared with that in the control group, the lipid droplet content in cells induced by OA+PA for 12 h and 24 h increased significantly (*p* < 0.001), while the lipid droplet content of those induced for 24 h was significantly higher than that for 12 h (*p* < 0.001), indicating that OA+PA could significantly increase the steatosis of AML-12, so the cells induced for 24 h were used to establish the MAFLD cell model in subsequent experiments.

### 2.2. Effects of Sch B on AML-12 Cell Activities

**Figure 2:**
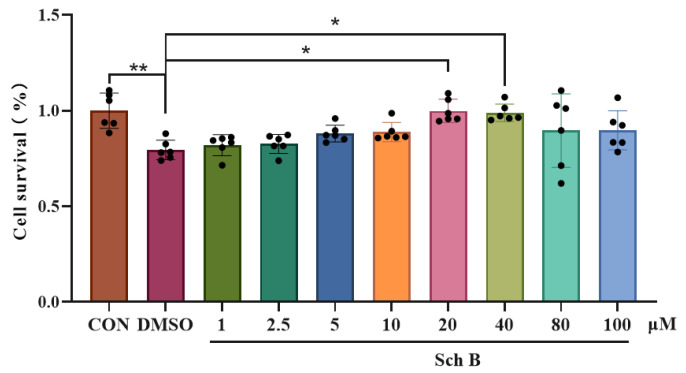
Effects of Sch B on AML-12 cell activities (mean ± *S.E.*, *n* = 6). * *p* < 0.05; ** *p* < 0.01.

The cell survival results showed that compared with those in the CON group, the OD values of cells in the DMSO group were significantly reduced (*p* < 0.01), and compared with those in the DMSO group, the OD values of cells in the 20 μM and 40 μM Sch B groups were significantly increased (*p* < 0.05), indicating that the inhibitory effect and toxicity of Sch B at these two concentrations on AML-12 cells were lower, so 20 and 40 μM SchB were selected for culturing AML-12 cells for 12 h in subsequent experiments (Figure 2).

### 2.3. Histomorphological Results of AML-12 Cells by Oil Red Staining

**Figure 3:**
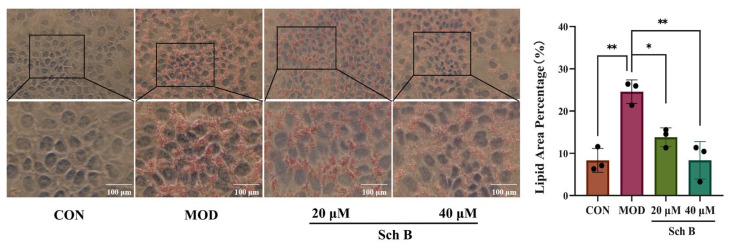
Histomorphological results of AML-12 cells stained with Oil Red (×100). (mean ± *S.E.*, *n* = 3). * *p* < 0.05; ** *p* < 0.01.

AML-12 cells were induced by OA+PA for 24 h to establish an MAFLD model, then 20 μM and 40 μM Sch B was added into the culture medium, respectively, and 12 h later, the cells were stained by Oil Red O staining. As shown in Figure 3, under a light microscope, the nuclei of AML-12 cells in the CON group were stained blue, and no red neutral fat color was observed, and those in the MOD group were stained blue, with an obvious red wrapping around the nucleus (vs. CON, *p* < 0.01), indicating that OA+PA could induce a large amount of neutral fat deposition in the cytoplasm, leading to cellular steatosis. Compared with those in the MOD group, the nuclei of AML12 cells in the 20 μM Sch B group showed only less red wrapping around the nucleus (vs. MOD, *p* < 0.05), while those in the 40 μM Sch B group exhibited much less red wrapping (vs. MOD, *p* < 0.01), indicating that the decrease in the concentration of Sch B could reduce the OAPA-induced lipid deposition in the cytoplasm, and the increase in the concentration of Sch B could alleviate the lipid deposition.

### 2.4. Effects of Sch B on TG, TC, ALT, and AST Levels in AML Cells

**Figure 4:**
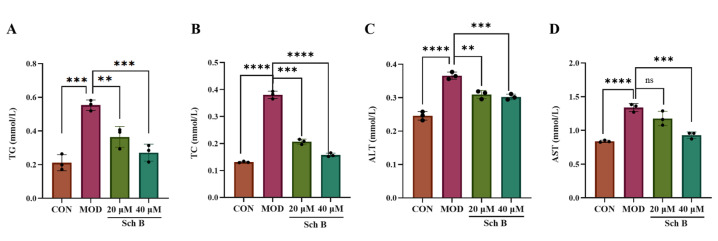
(**A**–**D**) Effects of Sch B on TG, TC, ALT, and AST levels in AML-12 cells (mean ± *S.E*., *n* = 3). ns (not significant); ** *p* < 0.01; *** *p* < 0.001; **** *p* < 0.0001.

As shown in Figure 4, the levels of TG, TC, ALT, and AST in the MOD group were significantly higher than those in the CON group (*p* < 0.001), and compared with those in the MOD group, the levels of TG, TC, ALT, and AST in the 20 and 40 μM Sch B groups were significantly lower (*p* < 0.01).

### 2.5. Network Pharmacology Prediction

#### 2.5.1. Acquisition of Intersecting Targets

**Figure 5:**
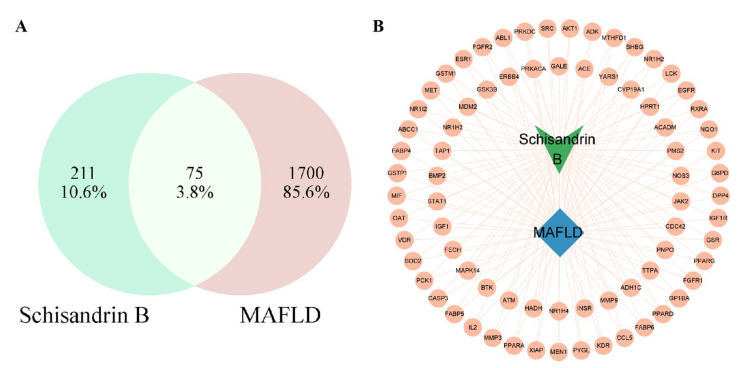
Venn diagram (**A**) and drug-disease target map (**B**) of Sch B and MAFLD-related targets.

The Sch B targets were predicted through the SwissTarget Prediction database, the CHEMBL database, and the PharmMapper database, and it was found that there were 286 targets of Sch B. With the keyword “metabolic-associated fat liver disease”, MAFLD-related targets were retrieved in the Genecards and OMIM databases. Using the Genecards database with a relevance score > 45 as the conditional card value, 1775 disease targets were obtained after they were merged and deduplicated. The target genes of Sch B and MAFLD were intersected, and then 75 intersection target genes were obtained, which were considered the interaction target genes for the drug treatment of MAFLD (Figure 5A). The “network. xlsx” and “type. xlsx” files were constructed using Sch B target data, and the files were imported into Cytoscape 3.9.1 to obtain a drug-disease target map (Figure 5B).

#### 2.5.2. Construction and Analysis of Protein–Protein Interaction Network (PPI)

**Figure 6:**
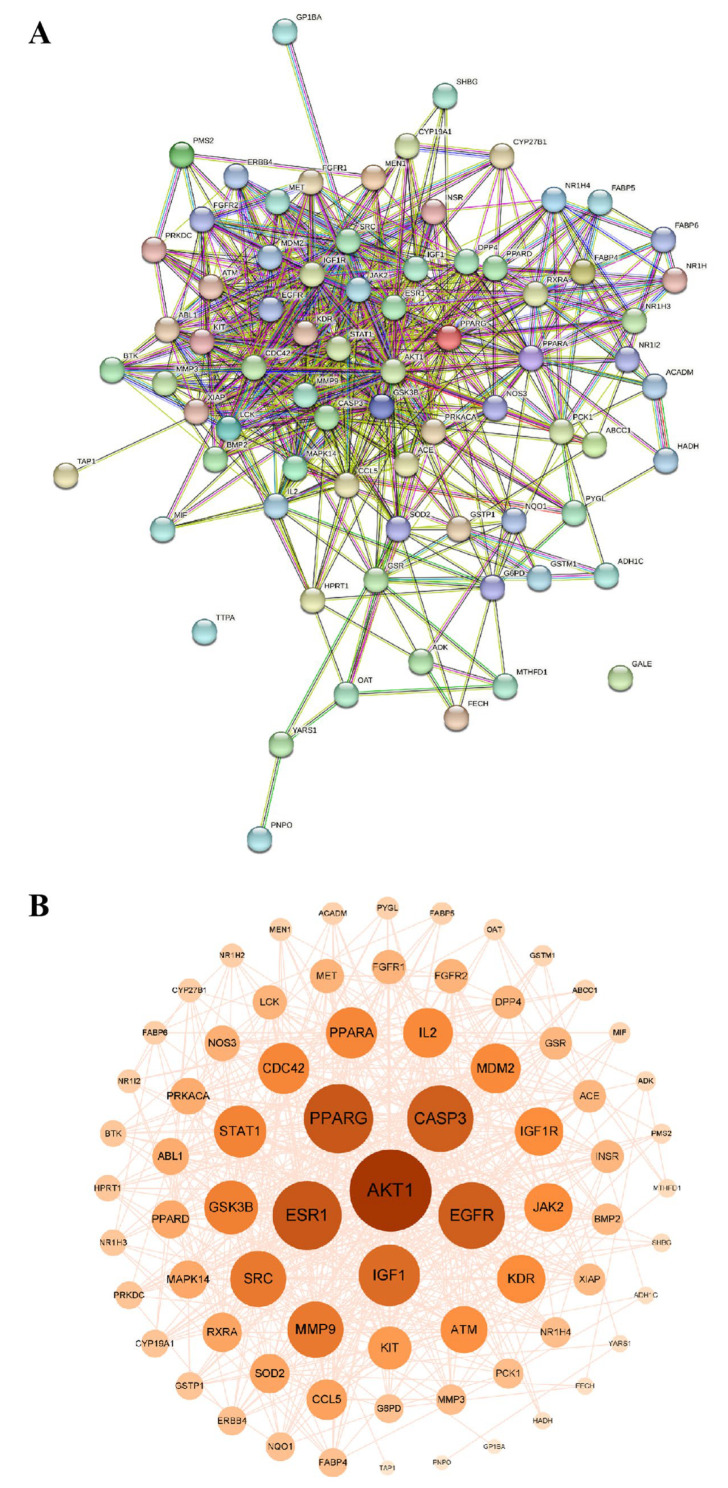
Protein–protein interaction network diagram. (**A**) Intersection target PPI network diagram; (**B**) core target network diagram.

The intersection gene file was imported into the STRING database to obtain a PPI. The centralized analysis and evaluation were conducted on all target gene information using Cytoscape 3.9.1. The median values of betweenness centrality (BC), compact centrality (CC), and degree centrality (DC) were set to be greater than or equal to the median for screening. The results showed that the median value of BC was 71.1232, that of CC was 0.0072, and that of DC was 16.4383, indicating that there were 14 nodes and 78 edges in the network. The top six screened targets were AKT1, ESR1, PPARG, CASP3, EGFR, and IGF1. Three core targets, PPARG, PCK-1, and CASP3, were identified through screening based on lipid metabolism and apoptosis (Figure 6).

#### 2.5.3. Gene Ontology (GO) Enrichment Analysis

**Figure 7:**
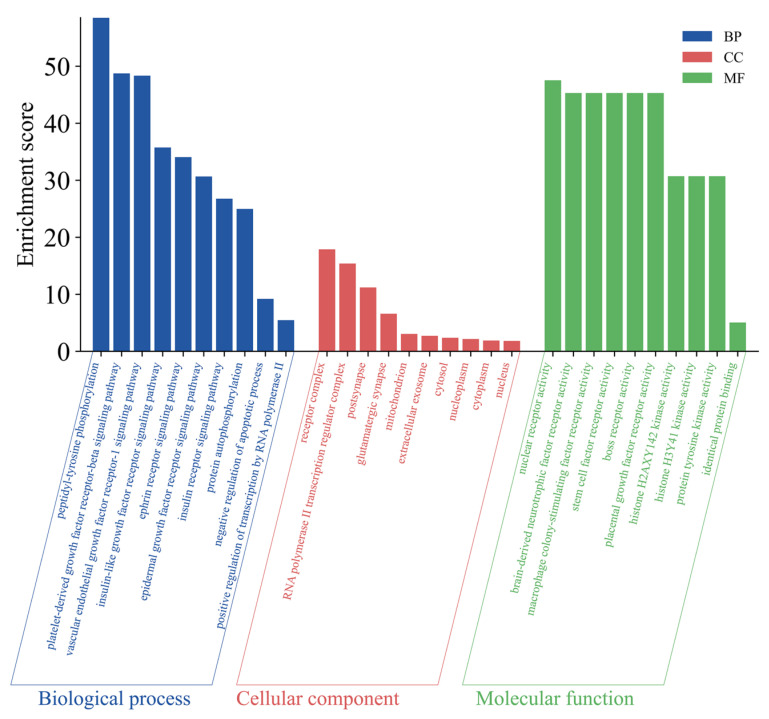
GO enrichment analysis bar chart.

The intersection targets were imported into the David database for enrichment analysis, and 405 GO entries were identified, of which 290 were related to biological processes (BP), mainly including peptidyl-tyrosine phosphorylation, platelet-derived growth factor receptor-beta signaling pathway, and vascular endothelial growth factor receptor-1 signaling pathway; 31 were related to cellular composition (CC), including receptor complex, RNA polymerase II transcription regulator complex, and postsynapse; 84 were related to molecular function (MF), including nuclear receptor activity, brain-derived neurotrophic factor receptor activity, and macrophage colony-stimulating factor receptor activity. The top 10 GO inputs of BP, CC, and MF were selected for plotting (Figure 7).

#### 2.5.4. Kyoto Encyclopedia of Genes and Genomes (KEGG) Pathway Enrichment Analysis

**Figure 8:**
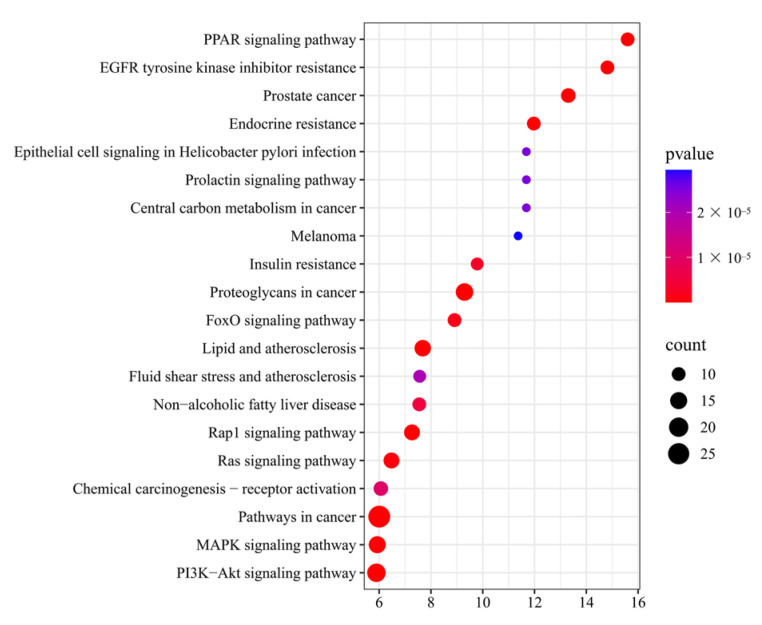
KEGG pathway enrichment bubble plot.

A total of 94 pathways were identified through KEGG pathway analysis, and 20 pathways were selected based on *p*-values. The top 20 KEGG pathways were plotted on a bubble plot (Figure 8), with Fold Enrichment values on the horizontal axis. The number of genes enriched in the corresponding pathways was represented by the size of the bubbles, and the depth of color represented significance, through which the significant enrichment information could be observed intuitively. Based on the three core targets of PPARG, PCK-1, and Caspase-3, the pathways involved in lipid metabolism and apoptosis mainly include the PPAR signaling pathway, lipid and atherosclerosis, and non-alcoholic fatty liver disease.

### 2.6. Molecular Docking Simulation Results

**Figure 9:**
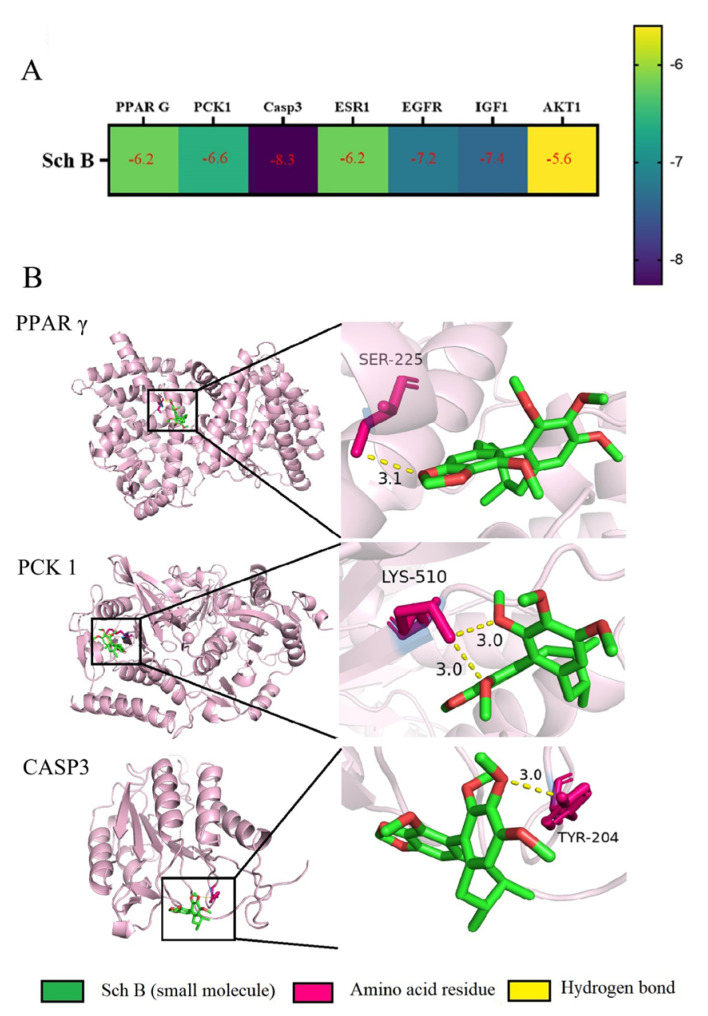
Molecular docking results of core targets. (**A**) Docking score. (**B**) Molecular docking diagram.

Molecular docking of Sch B with related core targets was performed using AutoDock Vina software (V1.2.7), and an energy matrix graph was plotted (the core components and core target values of Molecular docking are detailed in Supplementary Figure S1). The binding energy < −5.0 kJ/mol represents a good binding, and the visualization was achieved using PyMoL. The docking results showed that the binding energies of PCK-1, AKT1, ESR1, PPARG, CASP3, EGFR, and IGF1 were all < −5.0 kJ/mol (Figure 9A). Select lipid metabolism and apoptosis-related targets PPARG, PCK-1, and CASP3 were selected for visualization display (Figure 9B).

### 2.7. Expression of PPARγ Protein in MAFLD Cell Model

**Figure 10:**
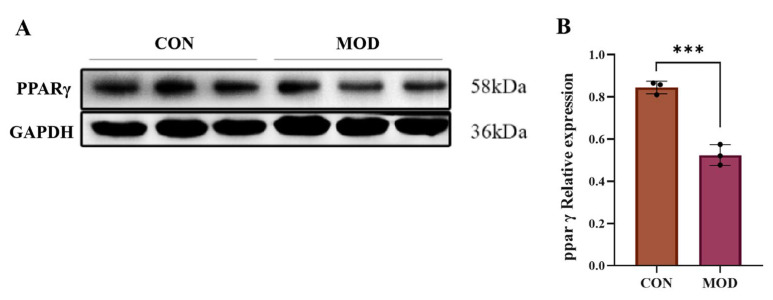
(**A**,**B**) Effect of OA+PA induction on PPARγ protein expression in AML-12 cells (mean ± *S.E.*, *n* = 3). *** *p* < 0.001.

The protein expression level of PPARγ in the AML-12 cell model of MAFLD mice induced by a high-fat diet (OA+PA) was detected by Western blot (Figure 10). It was found that compared with that in the CON group, the expression level of PPARγ protein in AML-12 cells in the MAFLD model group was significantly reduced (*p* < 0.001), indicating that PPARγ could play a crucial role in the progression of MAFLD.

### 2.8. Detection of PPAR γ Luciferase Reporter Gene

**Figure 11:**
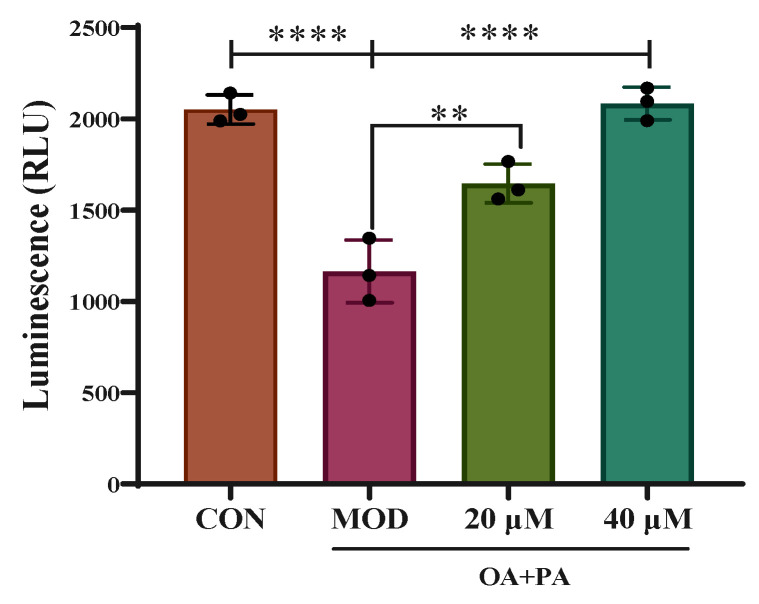
Results of PPARγ luciferase reporter gene (mean ± *S.E*., *n* = 3). ** *p* < 0.01; **** *p* < 0.0001.

As shown in Figure 11, the transcriptional activity of PPARγ in AML-12 cells decreased after OA+PA induction (CON vs. MOD, *p* < 0.01), while compared with that in the MOD group, the activity of PPARγ increased in the Sch B groups (20 μM Sch B vs. MOD, *p* < 0.01; 40 μM Sch B vs. MOD, *p* < 0.001).

### 2.9. Effects of Sch B on Expression of PPARγ Pathway-Related Proteins

**Figure 12:**
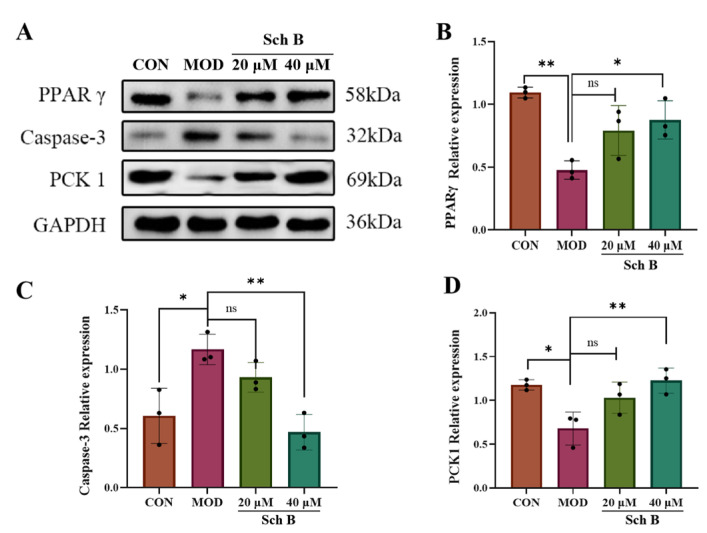
(**A**–**D**) Effects of Sch B on the expression of PPARγ, PCK1, and Caspase-3 proteins (mean ± *S.E*., *n* = 3). ns (not significant); * *p* < 0.05; ** *p* < 0.01.

Proteins involved in the regulation of lipid metabolism and apoptosis were screened by network pharmacology methods, including PPARγ, Caspase-3, and PCK1. The expression of these proteins was detected by Western blot, in which GAPDH was taken as an internal reference. As shown in Figure 12, compared with that in the CON group, the expression of PPARγ and PCK1 in the MOD group was significantly reduced (*p* < 0.01; *p* < 0.05), while the expression of Caspase-3 was significantly increased (*p* < 0.05); compared with that in the MOD group, the expression of PPARγ and PCK1 was significantly increased after Sch B treatment (*p* < 0.01; *p* < 0.05), while the expression of Caspase-3 was significantly decreased (*p* < 0.01).

### 2.10. Effects of Sch B on the Expression of Lipid Metabolism-Related Proteins

#### 2.10.1. Effects of Sch B on the Expression of Lipid Synthesis and Cholesterol Metabolism-Related Proteins

**Figure 13:**
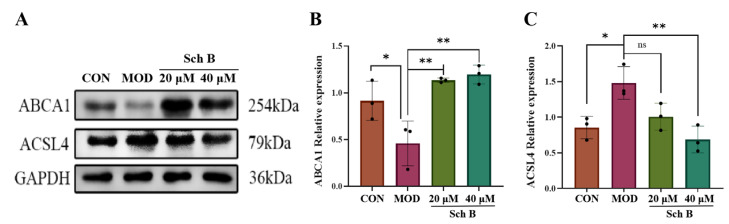
(**A**–**C**) Effects of Sch B on the expression of ABCA1 and ACSL4 proteins (mean ± *S.E.*, *n* = 3). ns (not significant); * *p* < 0.05; ** *p* < 0.01.

Compared with that in the CON group, the expression of cholesterol metabolism-related protein ABCA1 was significantly reduced in the MOD group (*p* < 0.05), indicating that the cholesterol reverse transport may be hindered and the fatty acid synthesis may be enhanced. Compared with that in the MOD group, the expression of ABCA1 in the Sch B groups was significantly increased (*p* < 0.01), suggesting that Sch B can regulate cholesterol metabolism-related proteins to promote cholesterol reverse transport, and inhibit the fatty acid synthesis. Compared with that in the CON group, the expression of fatty acid synthesis protein ACSL4 was significantly increased in the MOD group (*p* < 0.05), indicating an increase in fatty acid synthesis in the MOD group. Compared with that in the MOD group, the expression of histone was significantly reduced in the 40 μM Sch B group (*p* < 0.01), indicating that the intervention with Sch B could decrease the expression of fatty acid synthesis proteins, leading to a reduction in fatty acid synthesis (Figure 13).

#### 2.10.2. Fatty Acid Oxidation

**Figure 14:**
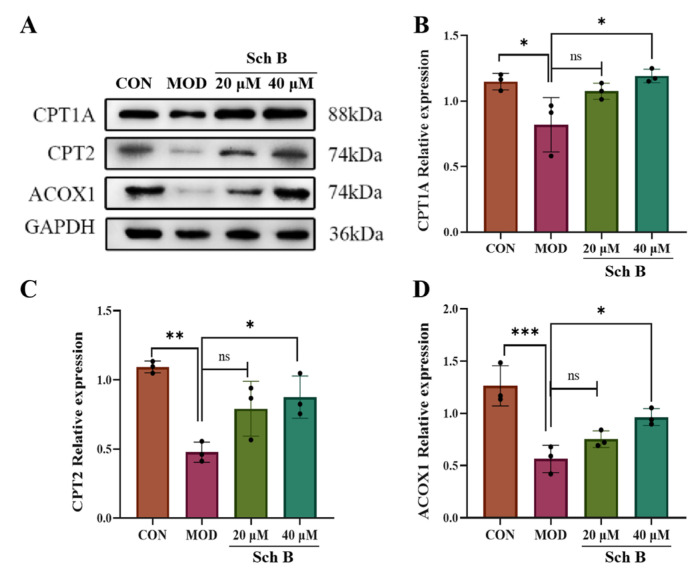
(**A**–**D**) Effects of Sch B on the expression of CPT1A, CPT2 and ACOX1 proteins (mean ± *S.E*., *n* = 3). ns (not significant); * *p* < 0.05; ** *p* < 0.01; *** *p* < 0.001.

Compared with those in the CON group, the expression levels of fatty acid oxidation-related proteins CPT1A, CPT2, and ACOX1 were significantly decreased in the MOD group (*p* < 0.05; *p* < 0.01; *p* < 0.001), indicating that the fatty acid β-oxidation process might be inhibited in the MOD group. Compared with those in the MOD group, the expression levels of these three proteins in the 40 μM Sch B group were significantly increased (*p* < 0.05), indicating that Sch B could promote the expression of fatty acid oxidation-related proteins and improve fatty acid oxidation function (Figure 14).

### 2.11. Effects of Sch B on Cell Apoptosis

#### 2.11.1. Annexin V-FITC/PI Detection of Cell Apoptosis

**Figure 15:**
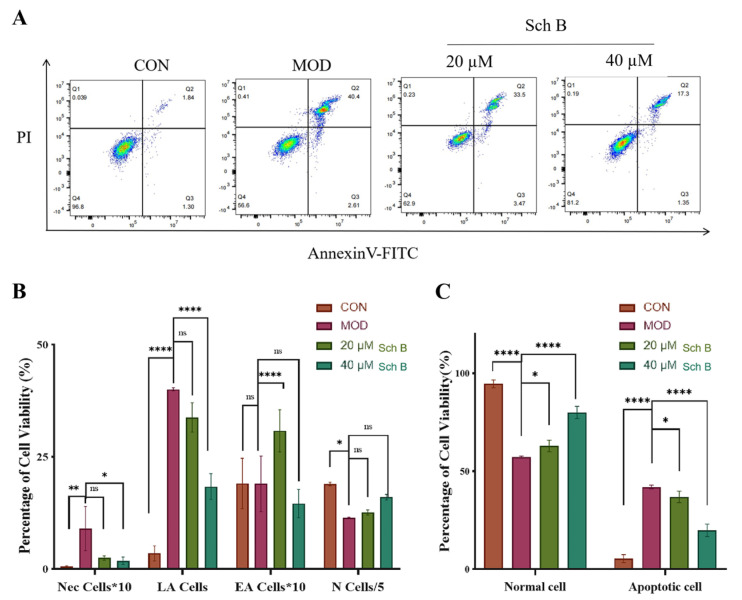
Effects of Sch B on apoptosis (mean ± *S.E*., *n* = 3). (**A**) Flow cytometry. (**B**) Flow cytometry visualization. (**C**) Comparison of healthy cells and apoptotic cells. ns (not significant); * *p* < 0.05; ** *p* < 0.01; **** *p* < 0.0001.

As shown in Figure 15, in the flow cytometry plot, the Q1 region (lower right) presents the distribution pattern of early apoptotic cells (EA cells), late apoptotic cells (LA cells) are identified and classified in the Q2 region (upper right), the Q3 region (lower left) exhibits normal cells (N cells), and mechanically damaged cells (Nec cells) are shown in the Q4 region (upper left).

The results showed that the proportion of late-stage apoptotic cells (LA cells) increased, while the proportion of normal cells (N cells) decreased in the MOD group, indicating the successful establishment of the apoptosis model. After the treatment with Sch B, the apoptosis of LA cells decreased gradually, while N cells rebounded, indicating that Sch B could partially inhibit apoptosis. The apoptotic cells in the 40 μM Sch B group were further reduced, while N cells recovered to near-normal cell levels, demonstrating a dose-dependent protective effect.

#### 2.11.2. Effects of Sch B on Expression of Apoptosis-Related Proteins

**Figure 16:**
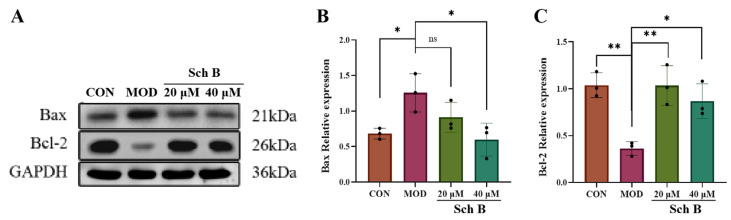
(**A**–**C**) Effects of Sch B on the expression of apoptosis-related proteins (mean ± *S.E*., *n* = 3). ns > 0.01; * *p* < 0.05; ** *p* < 0.01.

As shown in Figure 16, compared with that in the CON group, the relative expression level of pro-apoptotic protein Bax in the MOD group was significantly increased (*p* < 0.05), while the expression level of anti-apoptotic protein Bcl-2 was significantly decreased (*p* < 0.01). After the administration of Schi B, the relative expression levels of Bax and Bcl-2 proteins were significantly reversed compared to the MOD group, indicating that Sch B could significantly inhibit the expression of pro-apoptotic protein Bax and promote the expression of anti-apoptotic protein Bcl-2.

## 3. Discussion

From the perspective of cell model construction, oleic acid (OA) and palmitic acid (PA) are the two most commonly used free fatty acids that can simulate the pathological process of lipid accumulation in liver cells [21]. In this study, a mixed-induction MAFLD model of OA and PA (2:1) was used to simulate the pathological process of hepatic steatosis caused by the accumulation of free fatty acids in vivo. The Oil Red O staining results showed that after the induction for 24 h, the lipid droplet content was the highest, and the levels of TC, TG, and liver injury markers ALT and AST increased in AML cells, indicating a successful model construction. After the treatment with 20 μM and 40 μM Sch B, the lipid droplet deposition was dose-dependently reduced, and the levels of TC, TG, ALT, and AST were lowered in AML cells.

In terms of molecular mechanisms, 75 intersecting targets between Sch B and MAFLD was first screened by network pharmacology in this study, and the top 6 targets were further identified as AKT1, ESR1, PPARG, CASP3, EGFR, and IGF1 by constructing a PPI network diagram. Screening was conducted with a focus on lipid metabolism and apoptosis, among which PPARγ, PCK-1, and CASP3 were significantly enriched as core targets. PPARγ is widely expressed in various adipose tissues and participates in lipid metabolism activities, playing an important role in adipocyte differentiation, adipogenesis, lipid metabolism, and glucose metabolism [22]. On the other hand, Pck1, as a key enzyme in gluconeogenesis, plays a crucial regulatory role in liver energy metabolism, and its functional disorders are closely related to various metabolic diseases [23,24]. It is worth noting that our research team found in previous experiments that after the administration of Sch B, the upregulation of PPARγ in C57BL/6 mice could regulate the expression of its downstream protein Pck1 [20]. In addition, the Caspase family plays a crucial role in cell apoptosis [25]. An excessive accumulation of neutral lipids in the body can form lipid droplets, and free fatty acids that have not yet been metabolized in the body can deposit in the liver when the process of fatty acid oxidation is impaired [26]. Moreover, studies have shown that activating autophagy mechanisms can effectively improve lipid deposition in the body and simultaneously inhibit the process of cell apoptosis [27,28]. It was found in the KEGG pathway analysis that the activities of lipid metabolism-related pathways, such as PPAR signaling pathway, lipid and atherosclerosis, and non-alcoholic fatty liver disease, were high, indicating a research direction for subsequent mechanism studies. It was confirmed by the molecular docking simulation that the binding energy of Sch B to three lipid metabolism and apoptosis targets, PPARγ, PCK-1, and CASP3, was less than −5.0 kJ/mol, indicating the tight docking and strong affinity. In addition, the dual luciferase reporter plasmid detection directly confirmed that Sch B could directly activate the activity of PPARγ. Combined with Western blot to verify PPARγ and its downstream lipid metabolism and apoptosis genes, the experiment found that the expression of PPARγ and PCK-1 was significantly reduced and the expression of Caspase-3 was significantly increased in the MOD group. However, after Sch B treatment, the expression of PPARγ and its downstream lipid metabolism regulation-related genes (CPT1A, CPT2, ACOX1, ABCA1, ACSL4, and ABCA1) and apoptosis-related genes (Bax and Bcl-2) were reversed. The flow cytometry also showed that the administration of schisandrin B could alleviate the increase in late-stage apoptotic cells in MAFLD, with a dose-dependent protective effect.

Specifically, activating PPARγ is the “core switch” for Sch B to improve lipid metabolism disorders. On the one hand, PPARγ can reduce lipid synthesis at the source by inhibiting the expression of genes related to lipid synthesis. Acsl4, as a key enzyme regulating unsaturated fatty acid metabolism, is mainly distributed in peroxisomes and endoplasmic reticulum, playing an important catalytic role in fatty acid activation and participating in regulating fatty acid synthesis through transcriptional regulation mechanisms [29,30]. ABCA1 (ATP binding cassette transporter A1) is responsible for transporting intracellular cholesterol and phospholipids to the extracellular environment, which is related to the production of high-density lipoprotein (HDL), and involved in cholesterol reverse transport [31]. PPARγ inhibits lipid synthesis genes and promotes cholesterol reverse transport by activating ABCA1 simultaneously, reducing intracellular cholesterol levels to suppress lipid synthesis and improve lipid accumulation in liver cells. On the other hand, CPT1A, CPT2, and ACOX1 play important catalytic roles in the fatty acid β-oxidation metabolic pathway, among which CPT1A, as the main regulatory enzyme of this metabolic process, functions to transport long-chain fatty acids to the mitochondrial matrix for breakdown metabolism to generate energy [32], CPT2 is responsible for catalyzing the conversion reaction of acylcarnitine to acyl CoA, providing a substrate for subsequent oxidation processes [33], and ACOX1 mainly plays a key role in the oxidative degradation of extremely long-chain fatty acids [34]. PPARγ can directly regulate the expression of downstream fatty acid oxidation key enzymes such as CPT1A, CPT2, and ACOX1 to accelerate the entry of fatty acids into mitochondria for oxidative degradation and reduce lipid accumulation in liver cells. Bcl-2 and Bax proteins can determine the survival or apoptosis of cells. When the Bcl-2 protein is inhibited, its dimer with Bax decreases, leading to cell apoptosis [35]. PPARγ also plays a dominant role in inhibiting cell apoptosis [36]. In the pathological state of MAFLD, lipid accumulation may activate the apoptotic signaling pathway through the autophagy mechanism, and the activation of PPARγ can block the cascade reaction of liver cell apoptosis caused by MAFLD by downregulating Caspase-3 and Bax and upregulating Bcl-2 expression. It is speculated that Sch B may regulate the apoptosis-related proteins Bax and Bcl-2 by activating PPARγ, providing a protective barrier for liver cells and reducing cell death caused by lipid toxicity.

In summary, in this study, the potential targets of Sch B for treating MAFLD were predicted by network pharmacology analysis. The results showed that Sch B may regulate liver lipid metabolism and apoptosis through multiple targets and pathways, thereby exerting its therapeutic effects. It was confirmed that Sch B could specifically activate PPARγ as the core regulatory target to regulate the expression of downstream lipid metabolism and apoptosis-related genes, improving lipid metabolism disorders and inhibit cell apoptosis in OA+PA-induced MAFLD cell models, which may provide more direct and comprehensive experimental evidence for the interaction between Sch B and PPARγ, as well as the core regulatory role of PPARγ. In addition, the team’s previous study on Sch B alleviating MAFLD was further expanded in this study, in which a progressive study of “in vivo verification→in vitro mechanism analysis” was conducted. The in vivo efficacy of Sch B in a high-fat diet-induced mouse model had been confirmed previously by our team, while in this study, AML-12 liver cells were used for in vitro experiments to directly observe the effect of Sch B on liver cells. Then, the mechanism dimension of Sch B intervention in MAFLD was expanded by focusing on PCK1 and Caspase-3, in which PCK1, a key factor in metabolism, could provide a new direction for analyzing the regulation of Sch B on metabolic networks, and simultaneously, the study of Caspase-3-mediated cell apoptosis could answer the core question of how Sch B could protect liver cells from lipid toxicity-induced death. However, this study still has certain limitations. Network pharmacology has suggested multiple potential targets and pathways, but this study mainly focuses on PPARγ and its lipid metabolism and apoptosis-related genes, so the role of other pathways such as growth factor receptor signaling pathway and cancer in Sch B regulation has not been further studied. In the future, more experiments should be conducted to verify the synergistic effect of these pathways with PPARγ.

## 4. Materials and Methods

### 4.1. Experimental Drugs and Reagents

Sch B (Chengdu Pufide Biotechnology Co., Ltd., Chengdu, China); ALT, AST, TG, and TC reagent kits (Nanjing Jiancheng Bioengineering Research Institute, Nanjing, China); Modified Oil Red O staining kit (Shanghai Biyun Tian Biotechnology Co., Ltd., Shanghai, China); ECL chromogenic solution (Dalian Meilun Biotechnology Co., Ltd., Dalian, China); BCA protein assay kit, ammonium persulfate, 30% acrylamide, TEMED, 5×SDS loading buffer, glycine HCL-Tris, pancreatic enzymes, and streptomycin (Beijing Dingguo Changsheng Biotechnology Co., Ltd., Beijing, China); Skimmed milk powder (BD, Franklin Lakes, NJ, USA); Fetal bovine serum and DMEM/F12 culture medium (Shanghai Dathir Biotechnology Co., Ltd., Shanghai, China); Dual luciferase reporter gene detection kit (Promega, Madison, WI, USA); Annexin V-FITC/PI dual staining kit (Shanghai Beibo Biotechnology Co., Ltd., Shanghai, China); All antibodies (ABclonal, Wuhan, China).

### 4.2. Cytology Experiments

#### 4.2.1. AML-12 Cell Culture

AML-12 cells (Mouse cell line, Shanghai Instituteof Biochemistry and Cell Biology, Shanghai, China) were cultured in a complete medium containing DMEM/F12 medium + 10% FBS + 1% penicillin and streptomycin, in a 5% carbon dioxide incubator with a constant humidity and temperature at 37 °C. When the growth density of AML-12 cells reached about 90%, the cell passaging was performed.

#### 4.2.2. Establishment of MAFLD Cell Model

Palmitic acid (PA, 0.9188 g) and oleic acid (OA, 2.0094 g) powders were separately weighed and poured into centrifuge tubes, and then 50 mL of NaOH (100 mM) was added. The PA solution needed to be heated in an 80 °C water bath until the solution was clear. Then, 50 μL of the two solutions were separately added into 950 μL of BSA solution (10%) dropwise in a 55 °C water bath, shaken, and mixed repeatedly, and continued to be shaken and incubated for 10 min in a 55 °C water bath. At this time, the final concentration of PA was 3.3 mM, and that of OA was 6.6 mM. Finally, OA and PA solutions were filtered with a needle tube and a 0.22 mm filter in a super clean bench, and the filtered solutions were subpackaged into centrifuge tubes. AML-12 cells were incubated with the PA and OA solution in the complete culture medium at 37 °C for 24 h, and the cell solutions were stored in a refrigerator at 4 °C in a cell room, in which 100 µL OA and 100 µL PA were added to 1800 µL of complete culture medium.

### 4.3. CCK-8 Detection of Sch B Activity

An appropriate amount of Sch B was dissolved in DMSO to prepare a 100 mM stock solution. Then, 10 μL of 100 mM stock solution were mixed well with 990 μL of DMSO to prepare 1 mL of the solution at a concentration of 100 μM/L. The prepared 100 μM/L stock solution was diluted according to different concentration ratios, so that the final concentrations of Sch B were 1, 2.5, 5, 10, 20, 40, 80, and 100 μM/L, respectively. At the same time, a blank group and a DMSO control group were set up, and they were kept in a −20 °C refrigerator for packaging. AML-12 cells were inoculated into wells of a 96-well plate in advance, and then the old culture medium was discarded. The plate was washed with PBS, and the newly prepared complete culture medium was added into the well. Then, 1uL of stock solution containing different concentrations of Sch B was separately added into each well, with 6 duplicate wells in each group. The cells continued to be cultured in a cell incubator for 12 h, and then the cells were taken out. Following the instructions of the kit, 20 μL of detection reagent was added to each well, and the cells were incubated in the cell culture incubator for 2 h. OD values of the cell solutions at 450 nm were measured using an Infinite M200TECAN microplate reader (TECAN Group, Männedorf, Switzerland). Sch B concentrations for the subsequent experiments were screened and determined based on the cell survival rate results.

### 4.4. Detection of TG and TC

The counting of AML cells was performed under an optical microscope, and the cell density of AML cell suspension was adjusted to 1 million cells/mL. The prepared cell suspension was centrifuged at 1000 rpm for 10 min, and the supernatant was discarded to obtain the cell pellet. The cell pellet was dissolved in 0.2 mL of anhydrous ethanol for homogenization and sonicated under ice water bath conditions (power: 300 W for 3~5 s/time, at an interval of 30 s, and repeated 5 times) to prepare the cell homogenate. Then, 2.5 μL of cell homogenate and 250 μL of working solution were added into each well of a 96-well plate, while blank and calibration wells were set up and 3 duplicate wells were set up in each group. The well plate was shaken to mix the solution well, and the cells were incubated at 37 °C for 10 min. The absorbance value of the solution from each well was measured at the 500 nm wavelength using an enzyme-linked immunosorbent assay (ELISA) microplate reader. The contents of TG and TC in each group of cells were calculated based on the absolute OD value (measured well OD value minus control well OD value).

### 4.5. Determination of ALT and AST

The method used for preparing AML cells was the same as that in Section 4.4, and the wavelength for the measurement by enzyme-linked immunosorbent assay (ELISA) reader was set up at 505 nm for measuring the OD value of each well. The absolute OD values (measured well OD value minus control well OD value) were substituted into the standard curve to obtain the corresponding ALT and AST activity values.

### 4.6. Cell Oil Red O Staining Detection

AML cells were inoculated into a six-well plate in advance. The complete culture medium was discarded before staining, and the cells were washed three times with PBS, and fixed with 4% paraformaldehyde solution for 30 min. Then, the fixative was discarded, and the cells were washed twice with distilled water. The cells were soaked in 60% isopropanol for 5 min. The isopropanol was discarded, and newly prepared Oil Red O staining solution was added. After soaking for 20 min, the staining solution was discarded. The cells were washed with 60% isopropanol for 3 s, and then the isopropanol was discarded. The cells were rinsed with pure water 3 times until there was no excess staining solution. Mayer hematoxylin staining solution was added to redye the cell nucleus for 1 min, then the Mayer hematoxylin staining solution was discarded, and the cells were washed with water 3 times. After adding Oil Red O buffer, the cells were left standing for 1 min, and the buffer was discarded. Pure water was added to cover the cells, and the cells were observed and photographed under an optical microscope.

### 4.7. Prediction of Targets by Network Pharmacology Methods

#### 4.7.1. Acquisition of Intersection Targets of Sch B and MAFLD

The smile number of Sch B obtained from the Pubchem Database (https://pubchem.ncbi.nlm.nih.gov/, accessed on 7 April 2025) was imported into the Swiss Target Prediction Database (http://www.swisstargetprediction.ch/, accessed on 9 April 2025), the PharmMapper Database (https://www.lilab-ecust.cn/pharmmapper/submitfile.html, accessed on 9 April 2025), and the CHEMBL Database (https://www.ebi.ac.uk/chembl/, accessed on 10 April 2025) for the prediction of Sch B target genes, and the targets were standardized through the Uniprot Database (http://www.uniprot.org, accessed on 10 April 2025). Using the keyword ‘metabolic-associated fatty liver disease’, potential MAFLD targets were screened through GeneCards (http://www.Genecards.org/, accessed on 11 April 2025) and OMIM (https://omim.org/, accessed on 12 April 2025) Database and deduplicated. Sch B target and MAFLD targets were imported into the Wei Sheng Xin platform (http://www.bioinformatics.com.cn/, accessed on 13 April 2025), a bioinformatics platform, to obtain intersecting targets.

#### 4.7.2. Construction of Sch B Target Network

The intersection targets of Sch B and MAFLD were obtained through the Wei Sheng Xin Platform, and a Venn diagram was drawn for intuitive display. The interactions among Sch B, intersecting targets, and MAFLD were constructed and visualized using Cytoscape 3.9.1 software.

#### 4.7.3. Construction of Target PPI

PPI for Sch B was constructed through the STRING Database (http://www.string-db.org/, accessed on 15 April 2025). The obtained data were imported into Cytoscape 3.9.1, and the core target information was obtained by the screening based on the values of BC, CC, and DC larger than or equal to the median values.

#### 4.7.4. GO Biological Process Enrichment and KEGG Pathway Analysis

The intersection targets were imported into the David Database for GO (https://davidbioinformatics.nih.gov/, accessed on 18 April 2025) functional enrichment analysis and KEGG pathway enrichment analysis, and the results were visualized on the Wei Sheng Xin Visualization Cloud Platform.

### 4.8. Molecular Docking and Visualization Analysis

The molecular docking between Sch B and important targets was performed using AutoDock Vina software (V1.2.7), and its binding activity was verified. The 2D structure of Sch B was downloaded from the Pubchem Database and input into Chembio3D to minimize the energy, and then AutodockTools-1.5.7 was used for hydrogenation, calculation, and allocation of charges, and for setting rotation buttons, which were stored in the form of “pdbqt”. Uniprot ID was obtained from the Uniprot Database (http://www.uniprot.org/, accessed on 22 April 2025), and the key target proteins were downloaded through the RCSBPDB Database (https://www.rcsb.org/, accessed on 23 April 2025). The proteins were input into PyMoL software (V3.1.6.1) to remove the original ligands and water, and transferred into AutoDocktools (V1.5.7) for hydrogenation, calculation, and allocation of the charges, and for specifying the atomic type, which were stored in the form of “pdbqt”. The protein ligand was used as the center of the docking box, and the lattice box covered the proteins and molecules. Other parameters were default values. The interaction mode was studied using PyMOL technology.

### 4.9. Dual Luciferase Reporter Gene Detection

Following the instructions of luciferase reporter gene detection kit, the PPARγ luciferase reporter plasmid and lipofectamine 3000 mixed with serum-free Opti-MEM was incubated at room temperature for 20 min. This mixture was added to fetal bovine serum DMEM/F12 complete culture medium containing AML-12 cells, and the luciferase reporter gene construct and Renilla luciferase expression vector were co-transfected. Twenty-four hours later, the AML-12 cells were treated with Sch B for one hour, then incubated with OA+PA (1 μM) for two hours, and firefly and renilla luciferase activities were detected using a dual luciferase reporter gene assay kit. The PPARγ transcriptional activity was calculated as (relative light unit firefly luciferase/renilla luciferase) × 100.

### 4.10. Cell Apoptosis Detection by Annexin V-FITC/PI Dual Staining

Adherent AML-12 cells were digested with trypsin, followed by adding complete culture medium containing serum to terminate the digestion, and the cell suspension was centrifuged to collect the cell pellet. The cells were washed twice with pre-cooled PBS, and the cell suspension was centrifuged at 300 rpm for 5 min, and the supernatant was discarded to collect the cell pellet for preparing the cell suspension. Then, 100 μL of 1×Annexin V binding buffer were injected into the cell suspension, followed by adding 5 μL of Annexin V-FITC to it, which was incubated at room temperature in the dark for 15 min. The cell suspension was added with 5 μL of PI (the final concentration was 1–2 μg/mL), then incubated in the dark for 5 min, and immediately 400 μL of the binding buffer was added. The solution was mixed well for testing on the machine.

### 4.11. Protein Immunoblot Results

AML-12 cells were digested with trypsin and collected by centrifugation. The cells were washed twice with pre-cooled PBS solution, and lysed with RIPA lysis reagent at 4 °C for 30 min. The lysate was centrifuged at 12,000× *g* and 4 °C for 10 min to obtain the supernatant of cell lysate. The BCA protein quantification method was used to determine the total protein in the supernatant. To the protein supernatant 5 × SDS protein electrophoresis buffer was added, and then the cells were denatured in a boiling water bath for 5 min and stored at −20 °C for use. The proteins were separated by SDS-PAGE electrophoresis and transferred onto PVDF membranes for 2 h. The membranes were blocked with TBST containing 5% skim milk powder (1.5 h). The membranes were incubated with the primary antibodies at 4 °C overnight. Then, they were washed three times with TBST and incubated with the secondary antibody at room temperature for 1 h. A luminescent reagent was added on the membranes for development, and the images were photographed with a gel imager (UVP Chemsolo Auto gel imager, Jena Analytical Instruments, Jena, Germany). The grayscale values of different bands were measured using an Image J analysis software, in which GAPDH (1:50,000) was used as the internal reference. The ratio of each grayscale value to the GAPDH grayscale value was taken as the relative expression level of proteins.

### 4.12. Statistical Analysis

SigmaPlot 12.5 was used for analysis. The data results are presented in the form of mean ± standard error (mean ± S.E.), and Tukey’s tests were used to determine the level of inter-group significance. One way analysis of variance was used to evaluate the differences. Ns means *p* > 0.05, which indicated no statistical significance, whereas *p* < 0.05 indicated a statistical significance. GraphPad Prism 9.5.1 was used for plotting. The grayscale values detected by Western blot were analyzed using Image J software (V1.54g).

## 5. Conclusions

**Figure 17:**
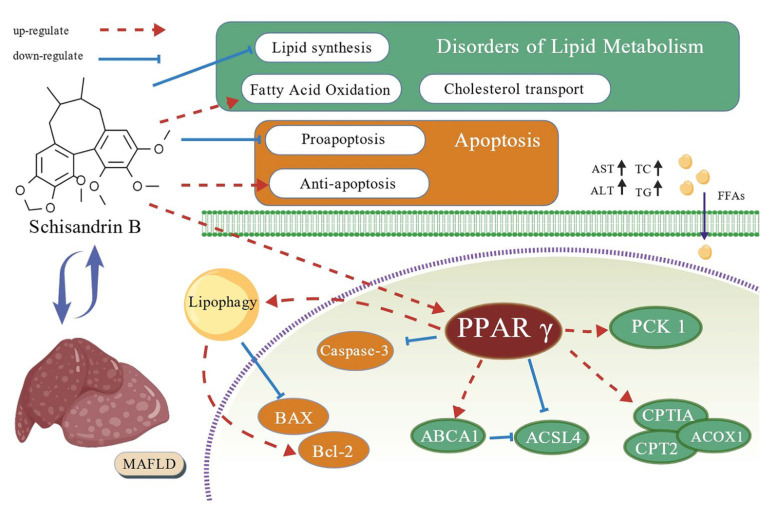
Potential mechanism of Sch B alleviating MAFLD.

An in vitro model of AML-12 mouse liver cell metabolism-related fatty liver disease (MAFLD) was constructed using oleic acid (OA) and palmitic acid (PA). The results showed that Schisandrin B has a significant regulatory effect on lipid metabolism disorders in the OA+PA-induced MAFLD cell model. Its mechanism of action mainly relies on the activation of the PPARγ-PCK1/Aspase-3 signaling pathway, which can not only inhibit the generation of fatty acids but also enhance the breakdown metabolism of fatty acids, thereby reducing lipid deposition in liver cells. Through this series of biochemical reactions, the lipid metabolism disorder in the MAFLD cell model could be significantly alleviated, and the process of cell apoptosis could be effectively inhibited (Figure 17). This finding may lay an important theoretical foundation and create a new research direction for the deep development and application of *Schisandra chinensis*.

## Data Availability

The original contributions presented in this study are included in the article/Supplementary Material. Further inquiries can be directed to the corresponding authors.

## Supplementary Materials

The following supporting information can be downloaded at: https://www.mdpi.com/article/10.3390/ph18101441/s1, Figure S1: Core components and core targets in molecular docking.

## Abbreviations

The following abbreviations are used in this manuscript:

Sch B	Schisandrin B
MAFLD	Metabolic-associated fatty liver disease
PPAγ	Peroxisome proliferator-activated receptor gamma
OA	Oleic acid
PA	Palmitic acid
TC	Total cholesterol
TG	Triglycerides
ALT	Alanine aminotransferase
AST	Aspartate aminotransferase
PPI	Protein–protein interaction network
BC	Betweenness centrality
CC	Compact centrality
DC	Degree centrality
GO	Gene Ontology
BP	Biological processes
MF	Molecular function
KEGG	Kyoto Encyclopedia of Genes and Genomes
