# Supplementary figures and images for: Schisandrin B Alleviates Lipid Metabolism Disorders and Apoptosis of MAFLD via Modulation of PPARγ-PCK1 and Caspase-3 Signaling Pathways
Source: Pharmaceuticals (Basel). 2025 Sep 25;18(10):1441. doi: 10.3390/ph18101441 (PMC12567207; doi:10.3390/ph18101441)

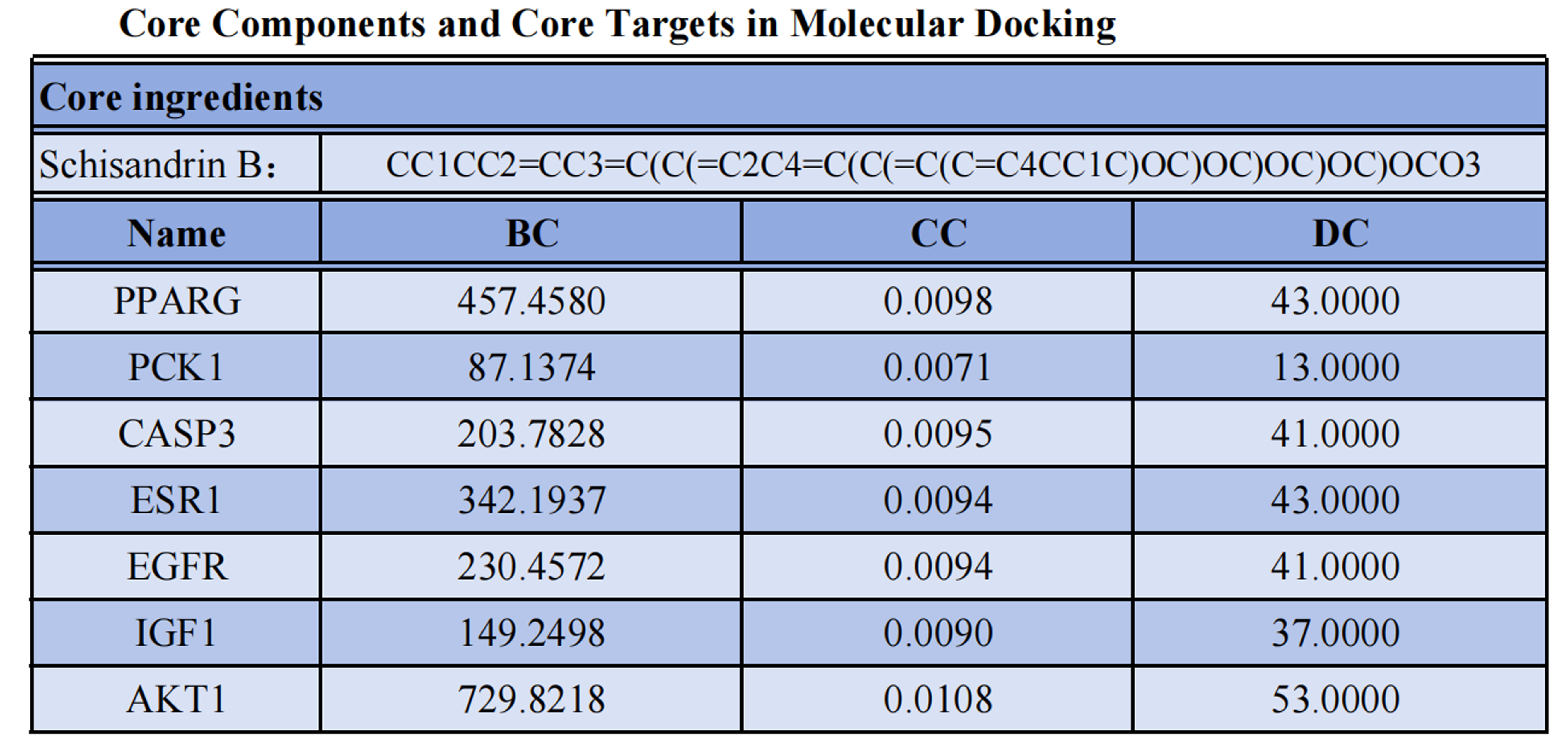

Supplement: Supplementary file 1 [file pharmaceuticals-18-01441-s001.zip › pharmaceuticals-3862247-supplementary.tif]
